# Bis(benzothia­zol-2-ylmeth­yl)amine

**DOI:** 10.1107/S1600536809023435

**Published:** 2009-06-24

**Authors:** Yong Zhang, Bi-lin Zhao, Shi-lei Zhang, Yuan Qu, Xian-you Xia

**Affiliations:** a School of Chemical and Materials Engineering, Huangshi Institute of Technology, Huangshi 435003, People’s Republic of China

## Abstract

In the title compound, C_16_H_13_N_3_S_2_, the dihedral angle between the two benzothia­zole ring systems is 20.41 (2)°. In the crystal structure, inter­molecular N—H⋯N hydrogen bonds link mol­ecules into a chain along the *b* axis. The packing is further stabilized by C—H⋯π stacking inter­actions involving the two benzothia­zole ring systems.

## Related literature

For applications of benzothiazole devivatives, see: Pinheiro *et al.* (1990 ▶); Emad *et al.* (2009 ▶).  For their use as ligands, see: Oughtred *et al.* (1982 ▶); Akther *et al.* (2008 ▶). For related structures, see: Laurence *et al.* (1980 ▶.
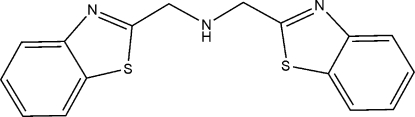


## Experimental

### 

#### Crystal data

C_16_H_13_N_3_S_2_
*M* *_r_* = 311.41
Monoclinic, 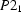

*a* = 7.8478 (5) Å
*b* = 5.8042 (3) Å
*c* = 16.1548 (9) Å
β = 97.910 (1)°
*V* = 728.85 (7) Å^3^
*Z* = 2
Mo *K*α radiation
μ = 0.36 mm^−1^
*T* = 298 K
0.23 × 0.12 × 0.10 mm

#### Data collection

Bruker SMART APEX CCD area-detector diffractometer
Absorption correction: multi-scan (*SADABS*; Bruker, 2001 ▶) *T*_min_ = 0.889, *T*_max_ = 0.965
9009 measured reflections
3603 independent reflections
3473 reflections with *I* > 2σ(*I*)
*R*_int_ = 0.032

#### Refinement

*R*[*F* ^2^ > 2σ(*F* ^2^)] = 0.029
*wR*(*F* ^2^) = 0.079
*S* = 1.07
3603 reflections
193 parameters
1 restraint
H atoms treated by a mixture of independent and constrained refinement
Δρ_max_ = 0.19 e Å^−3^
Δρ_min_ = −0.22 e Å^−3^
Absolute structure: Flack (1983 ▶), 1623 Friedel pairs
Flack parameter: −0.07 (4)

### 

Data collection: *SMART* (Bruker, 2001 ▶); cell refinement: *SAINT-Plus* (Bruker, 2001 ▶; data reduction: *SAINT-Plus* program(s) used to solve structure: *SHELXS97* (Sheldrick, 2008 ▶); program(s) used to refine structure: *SHELXL97* (Sheldrick, 2008 ▶); molecular graphics: *PLATON* (Spek, 2009 ▶); software used to prepare material for publication: *SHELXTL* (Sheldrick, 2008 ▶).

## Supplementary Material

Crystal structure: contains datablocks global, I. DOI: 10.1107/S1600536809023435/pk2169sup1.cif

Structure factors: contains datablocks I. DOI: 10.1107/S1600536809023435/pk2169Isup2.hkl

Additional supplementary materials:  crystallographic information; 3D view; checkCIF report

## Figures and Tables

**Table 1:** Hydrogen-bond geometry (Å, °)

*D*—H⋯*A*	*D*—H	H⋯*A*	*D*⋯*A*	*D*—H⋯*A*
N1—H1⋯N2^i^	0.821 (19)	2.489 (19)	3.3054 (18)	173.5 (17)
C1—H1⋯*Cg*1^ii^	0.97	2.78	3.737 (16)	168
C9—H9⋯*Cg*2^ii^	0.97	2.73	3.689 (17)	170
C14—H14⋯*Cg*3^iii^	0.93	2.89	3.598 (2)	134

Symmetry codes: (i) 
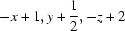
; (ii) 
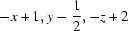
; (iii) 
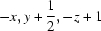
. *Cg*1, *Cg*2, *Cg*3 are the centroids of the S1,C2,N2,C3,C8, C3–C8 and C11–C16 rings, respectively.

**Fig. 1.**
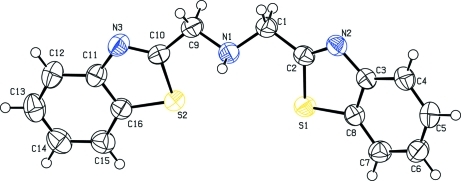
The molecular structure with displacement ellipsoids drawn at the 50% probability level.

**Fig. 2.**
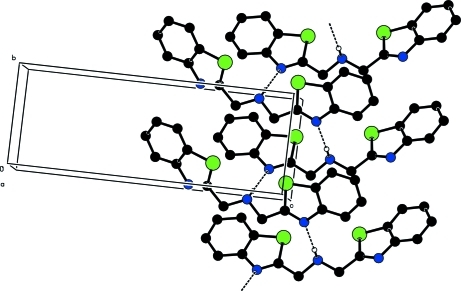
Part of the crystal structure showing hydrogen bonds as dashed lines.

## supplementary crystallographic information

### Comment

Benzothiazole devivatives have been used as photostablizers and metal chelating agents (Pinheiro *et al.*, 1990; Emad *et al.*, 2009). Many chelating heterocyclic ligands bearing benzothiazole group have been reported in recent years (Oughtred *et al.*, 1982; Akther *et al.*, 2008).  The wide range of application of the benzothiazole chelators and their metal complexes aroused our interest to prepare a new series of metal complexes. With this mind, the title compound was prepared and we report the crystal stucture herein.

In the molecular structure (Fig. 1), the dihedral angle between the two benzothiazole ring systems is 20.41 (2)°. The C—N bond distances range from 1.2906 (18) to 1.4567 (18) Å, and the *C*—*N*(amino) bonds are longer than the C—N (benzothiazolyl) bonds. In the crystal structure (Fig. 2), intermolecular N—H···N hydrogen bond links molecules into a chain along the *b* axis. The packing is further stabilized by C—H···π stacking interactions involving two benzothiazole ring systems.

### Experimental

The title compound was synthesized according to a literature procedure (Laurence *et al.*, 1980). Single crystals suitable for X-ray diffraction were obtained by slow evaporation of a dichloromethane solution at room temperature.

### Refinement

H atoms bonded to carbon atoms were placed in idealized positions [*C*—*H*(methylene)=0.97 Å and C—H(aromatic) =0.93 Å] and included in therefinement in the riding-model approximation, with *U*_iso_(methyl and aromatic H) = 1.2Ueq(C). H atoms bonded to N atom was found from the difference map and refined with the restraint of N—H=0.86 (1)Å and *U*_iso_(H)=1.2*U*_eq_(N).

### Crystal data

**Table:**

C_16_H_13_N_3_S_2_	*F*(000) = 324
*M**_r_* = 311.41	*D*_x_ = 1.419 Mg m^−^^3^
Monoclinic, *P*2_1_	Mo *K*α radiation, λ = 0.71073 Å
Hall symbol: P 2yb	Cell parameters from 6428 reflections
*a* = 7.8478 (5) Å	θ = 2.6–28.2°
*b* = 5.8042 (3) Å	µ = 0.36 mm^−^^1^
*c* = 16.1548 (9) Å	*T* = 298 K
β = 97.910 (1)°	Block, colourless
*V* = 728.85 (7) Å^3^	0.23 × 0.12 × 0.10 mm
*Z* = 2	

### Data collection

**Table:**

Bruker SMART APEX CCD area-detector diffractometer	3603 independent reflections
Radiation source: fine focus sealed Siemens Mo tube	3473 reflections with *I* > 2σ(*I*)
graphite	*R*_int_ = 0.032
0.3° wide ω exposures scans	θ_max_ = 28.3°, θ_min_ = 2.6°
Absorption correction: multi-scan (*SADABS*; Sheldrick, 2001)	*h* = −10→10
*T*_min_ = 0.889, *T*_max_ = 0.965	*k* = −7→7
9009 measured reflections	*l* = −21→21

### Refinement

**Table:**

Refinement on *F*^2^	Secondary atom site location: difference Fourier map
Least-squares matrix: full	Hydrogen site location: inferred from neighbouring sites
*R*[*F*^2^ > 2σ(*F*^2^)] = 0.029	H atoms treated by a mixture of independent and constrained refinement
*wR*(*F*^2^) = 0.079	*w* = 1/[σ^2^(*F*_o_^2^) + (0.0549*P*)^2^ + 0.0014*P*] where *P* = (*F*_o_^2^ + 2*F*_c_^2^)/3
*S* = 1.07	(Δ/σ)_max_ = 0.002
3603 reflections	Δρ_max_ = 0.19 e Å^−^^3^
193 parameters	Δρ_min_ = −0.22 e Å^−^^3^
1 restraint	Absolute structure: Flack (1983), 1621 Friedel pairs
Primary atom site location: structure-invariant direct methods	Flack parameter: −0.07 (4)

### Special details

**Table:**

Geometry. All e.s.d.'s (except the e.s.d. in the dihedral angle between two l.s. planes) are estimated using the full covariance matrix. The cell e.s.d.'s are taken into account individually in the estimation of e.s.d.'s in distances, angles and torsion angles; correlations between e.s.d.'s in cell parameters are only used when they are defined by crystal symmetry. An approximate (isotropic) treatment of cell e.s.d.'s is used for estimating e.s.d.'s involving l.s. planes.
Refinement. Refinement of *F*^2^ against ALL reflections. The weighted *R*-factor *wR* and goodness of fit *S* are based on *F*^2^, conventional *R*-factors *R* are based on *F*, with *F* set to zero for negative *F*^2^. The threshold expression of *F*^2^ > σ(*F*^2^) is used only for calculating *R*-factors(gt) *etc*. and is not relevant to the choice of reflections for refinement. *R*-factors based on *F*^2^ are statistically about twice as large as those based on *F*, and *R*- factors based on ALL data will be even larger.

### Fractional atomic coordinates and isotropic or equivalent isotropic displacement parameters (Å^2^)

**Table:**

	*x*	*y*	*z*	*U*_iso_*/*U*_eq_	
C1	0.32522 (18)	−0.2480 (3)	0.92805 (8)	0.0469 (3)	
H1A	0.4335	−0.3306	0.9380	0.056*	
H1B	0.2355	−0.3573	0.9078	0.056*	
C2	0.28685 (17)	−0.1421 (3)	1.00759 (9)	0.0421 (3)	
C3	0.27501 (17)	−0.0905 (3)	1.14199 (9)	0.0419 (3)	
C4	0.30208 (19)	−0.1344 (3)	1.22742 (9)	0.0508 (3)	
H4	0.3569	−0.2685	1.2483	0.061*	
C5	0.2453 (2)	0.0270 (3)	1.28049 (10)	0.0563 (4)	
H5	0.2626	0.0001	1.3378	0.068*	
C6	0.1634 (2)	0.2274 (4)	1.25053 (10)	0.0552 (4)	
H6	0.1279	0.3328	1.2880	0.066*	
C7	0.13337 (18)	0.2734 (3)	1.16584 (9)	0.0512 (3)	
H7	0.0775	0.4074	1.1456	0.061*	
C8	0.18981 (17)	0.1116 (3)	1.11172 (8)	0.0430 (3)	
C9	0.3518 (2)	−0.1629 (3)	0.78314 (9)	0.0520 (4)	
H9A	0.2734	−0.2916	0.7712	0.062*	
H9B	0.4680	−0.2194	0.7827	0.062*	
C10	0.31251 (18)	0.0167 (3)	0.71708 (9)	0.0440 (3)	
C11	0.30569 (18)	0.1982 (3)	0.59715 (9)	0.0463 (3)	
C12	0.3417 (2)	0.2449 (4)	0.51678 (10)	0.0589 (4)	
H12	0.4135	0.1486	0.4912	0.071*	
C13	0.2688 (2)	0.4370 (4)	0.47583 (10)	0.0628 (5)	
H13	0.2930	0.4705	0.4224	0.075*	
C14	0.1602 (2)	0.5812 (4)	0.51276 (11)	0.0617 (4)	
H14	0.1131	0.7100	0.4840	0.074*	
C15	0.1213 (2)	0.5352 (3)	0.59197 (11)	0.0571 (4)	
H15	0.0482	0.6313	0.6168	0.069*	
C16	0.19401 (18)	0.3417 (3)	0.63367 (9)	0.0449 (3)	
N1	0.33530 (16)	−0.0701 (2)	0.86544 (8)	0.0462 (3)	
H1	0.413 (2)	0.023 (3)	0.8796 (11)	0.055*	
N2	0.32946 (14)	−0.2309 (2)	1.08061 (7)	0.0448 (3)	
N3	0.37321 (16)	0.0144 (3)	0.64678 (8)	0.0499 (3)	
S1	0.17864 (5)	0.12218 (6)	1.00381 (2)	0.04752 (10)	
S2	0.17133 (5)	0.23922 (7)	0.73265 (2)	0.05010 (11)	

### Atomic displacement parameters (Å^2^)

**Table:**

	*U*^11^	*U*^22^	*U*^33^	*U*^12^	*U*^13^	*U*^23^
C1	0.0526 (7)	0.0424 (7)	0.0455 (7)	0.0054 (7)	0.0062 (6)	0.0017 (6)
C2	0.0395 (6)	0.0388 (7)	0.0475 (7)	0.0010 (5)	0.0043 (5)	0.0032 (6)
C3	0.0399 (6)	0.0417 (7)	0.0443 (7)	−0.0007 (5)	0.0062 (5)	0.0049 (5)
C4	0.0519 (8)	0.0532 (8)	0.0471 (8)	0.0010 (7)	0.0059 (6)	0.0091 (7)
C5	0.0587 (9)	0.0677 (10)	0.0437 (8)	−0.0003 (8)	0.0109 (7)	0.0042 (7)
C6	0.0572 (8)	0.0576 (9)	0.0528 (8)	0.0004 (8)	0.0145 (6)	−0.0076 (8)
C7	0.0522 (8)	0.0476 (8)	0.0536 (8)	0.0050 (7)	0.0065 (6)	0.0001 (7)
C8	0.0416 (6)	0.0446 (7)	0.0425 (6)	−0.0012 (6)	0.0044 (5)	0.0024 (6)
C9	0.0669 (9)	0.0443 (8)	0.0453 (8)	0.0058 (7)	0.0093 (7)	−0.0039 (6)
C10	0.0469 (6)	0.0413 (7)	0.0435 (7)	0.0005 (6)	0.0046 (5)	−0.0077 (6)
C11	0.0440 (7)	0.0546 (9)	0.0392 (6)	−0.0016 (6)	0.0022 (5)	−0.0045 (6)
C12	0.0568 (8)	0.0778 (12)	0.0426 (7)	0.0046 (9)	0.0082 (6)	−0.0028 (9)
C13	0.0641 (9)	0.0788 (13)	0.0439 (8)	−0.0109 (9)	0.0011 (7)	0.0083 (8)
C14	0.0631 (9)	0.0596 (11)	0.0592 (9)	−0.0012 (8)	−0.0036 (7)	0.0124 (8)
C15	0.0592 (8)	0.0528 (9)	0.0589 (9)	0.0037 (8)	0.0059 (7)	0.0034 (8)
C16	0.0432 (7)	0.0445 (7)	0.0469 (7)	−0.0045 (6)	0.0055 (5)	−0.0024 (6)
N1	0.0540 (7)	0.0441 (7)	0.0405 (6)	−0.0066 (6)	0.0063 (5)	−0.0029 (5)
N2	0.0470 (6)	0.0423 (6)	0.0454 (6)	0.0026 (5)	0.0079 (5)	0.0056 (5)
N3	0.0542 (6)	0.0545 (7)	0.0411 (6)	0.0064 (6)	0.0066 (5)	−0.0048 (6)
S1	0.0553 (2)	0.04359 (19)	0.04214 (17)	0.00959 (16)	0.00134 (13)	0.00366 (15)
S2	0.0592 (2)	0.04371 (19)	0.05053 (19)	0.00410 (16)	0.01872 (15)	−0.00142 (15)

### Geometric parameters (Å, °)

**Table:**

C1—N1	1.4554 (19)	C9—C10	1.493 (2)
C1—C2	1.4922 (19)	C9—H9A	0.9700
C1—H1A	0.9700	C9—H9B	0.9700
C1—H1B	0.9700	C10—N3	1.2906 (18)
C2—N2	1.2884 (18)	C10—S2	1.7424 (15)
C2—S1	1.7503 (15)	C11—C12	1.393 (2)
C3—C4	1.391 (2)	C11—N3	1.394 (2)
C3—N2	1.3951 (18)	C11—C16	1.397 (2)
C3—C8	1.404 (2)	C12—C13	1.380 (3)
C4—C5	1.384 (2)	C12—H12	0.9300
C4—H4	0.9300	C13—C14	1.387 (3)
C5—C6	1.383 (3)	C13—H13	0.9300
C5—H5	0.9300	C14—C15	1.382 (2)
C6—C7	1.382 (2)	C14—H14	0.9300
C6—H6	0.9300	C15—C16	1.391 (2)
C7—C8	1.396 (2)	C15—H15	0.9300
C7—H7	0.9300	C16—S2	1.7382 (15)
C8—S1	1.7344 (13)	N1—H1	0.821 (19)
C9—N1	1.4567 (18)		
			
N1—C1—C2	110.05 (13)	C10—C9—H9B	109.4
N1—C1—H1A	109.7	H9A—C9—H9B	108.0
C2—C1—H1A	109.7	N3—C10—C9	123.85 (14)
N1—C1—H1B	109.7	N3—C10—S2	116.97 (13)
C2—C1—H1B	109.7	C9—C10—S2	119.14 (11)
H1A—C1—H1B	108.2	C12—C11—N3	125.12 (15)
N2—C2—C1	124.47 (14)	C12—C11—C16	119.78 (16)
N2—C2—S1	116.49 (12)	N3—C11—C16	115.10 (12)
C1—C2—S1	119.04 (11)	C13—C12—C11	118.72 (17)
C4—C3—N2	125.36 (14)	C13—C12—H12	120.6
C4—C3—C8	119.93 (14)	C11—C12—H12	120.6
N2—C3—C8	114.70 (12)	C12—C13—C14	121.34 (16)
C5—C4—C3	118.20 (15)	C12—C13—H13	119.3
C5—C4—H4	120.9	C14—C13—H13	119.3
C3—C4—H4	120.9	C15—C14—C13	120.60 (17)
C6—C5—C4	121.73 (15)	C15—C14—H14	119.7
C6—C5—H5	119.1	C13—C14—H14	119.7
C4—C5—H5	119.1	C14—C15—C16	118.44 (16)
C7—C6—C5	121.09 (17)	C14—C15—H15	120.8
C7—C6—H6	119.5	C16—C15—H15	120.8
C5—C6—H6	119.5	C15—C16—C11	121.09 (14)
C6—C7—C8	117.68 (16)	C15—C16—S2	129.40 (13)
C6—C7—H7	121.2	C11—C16—S2	109.50 (11)
C8—C7—H7	121.2	C1—N1—C9	113.07 (13)
C7—C8—C3	121.37 (13)	C1—N1—H1	112.4 (13)
C7—C8—S1	128.98 (12)	C9—N1—H1	110.0 (12)
C3—C8—S1	109.60 (11)	C2—N2—C3	110.55 (13)
N1—C9—C10	111.02 (13)	C10—N3—C11	109.95 (13)
N1—C9—H9A	109.4	C8—S1—C2	88.66 (7)
C10—C9—H9A	109.4	C16—S2—C10	88.47 (7)
N1—C9—H9B	109.4		
			
N1—C1—C2—N2	−153.63 (14)	C12—C11—C16—C15	−1.8 (2)
N1—C1—C2—S1	25.69 (16)	N3—C11—C16—C15	178.18 (14)
N2—C3—C4—C5	177.62 (14)	C12—C11—C16—S2	179.13 (13)
C8—C3—C4—C5	−0.9 (2)	N3—C11—C16—S2	−0.91 (16)
C3—C4—C5—C6	0.1 (2)	C2—C1—N1—C9	−173.01 (13)
C4—C5—C6—C7	0.6 (3)	C10—C9—N1—C1	164.09 (13)
C5—C6—C7—C8	−0.5 (2)	C1—C2—N2—C3	−179.90 (13)
C6—C7—C8—C3	−0.4 (2)	S1—C2—N2—C3	0.76 (16)
C6—C7—C8—S1	−177.64 (12)	C4—C3—N2—C2	−179.17 (14)
C4—C3—C8—C7	1.1 (2)	C8—C3—N2—C2	−0.58 (18)
N2—C3—C8—C7	−177.61 (13)	C9—C10—N3—C11	176.92 (14)
C4—C3—C8—S1	178.83 (11)	S2—C10—N3—C11	−0.93 (17)
N2—C3—C8—S1	0.15 (15)	C12—C11—N3—C10	−178.86 (15)
N1—C9—C10—N3	154.02 (15)	C16—C11—N3—C10	1.18 (18)
N1—C9—C10—S2	−28.18 (18)	C7—C8—S1—C2	177.75 (14)
N3—C11—C12—C13	−178.31 (16)	C3—C8—S1—C2	0.21 (10)
C16—C11—C12—C13	1.7 (2)	N2—C2—S1—C8	−0.59 (11)
C11—C12—C13—C14	−0.6 (3)	C1—C2—S1—C8	−179.96 (12)
C12—C13—C14—C15	−0.3 (3)	C15—C16—S2—C10	−178.68 (16)
C13—C14—C15—C16	0.2 (3)	C11—C16—S2—C10	0.31 (11)
C14—C15—C16—C11	0.8 (2)	N3—C10—S2—C16	0.37 (13)
C14—C15—C16—S2	179.72 (13)	C9—C10—S2—C16	−177.58 (13)

### Hydrogen-bond geometry (Å, °)

**Table:**

*D*—H···*A*	*D*—H	H···*A*	*D*···*A*	*D*—H···*A*
N1—H1···N2^i^	0.821 (19)	2.489 (19)	3.3054 (18)	173.5 (17)
C1—H1···Cg1^ii^	0.97	2.78	3.737 (16)	168
C9—H9···Cg2^ii^	0.97	2.73	3.689 (17)	170
C14—H14···Cg3^iii^	0.93	2.89	3.598 (2)	134

Symmetry codes: (i) −*x*+1, *y*+1/2, −*z*+2; (ii) −*x*+1, *y*−1/2, −*z*+2; (iii) −*x*, *y*+1/2, −*z*+1.
